# Pattern of antibody responses to *Plasmodium falciparum* antigens in individuals differentially exposed to *Anopheles* bites

**DOI:** 10.1186/s12936-020-03160-5

**Published:** 2020-02-21

**Authors:** Kakou G. Aka, Dipomin F. Traoré, André B. Sagna, Dounin D. Zoh, Serge B. Assi, Bertin N’cho Tchiekoi, Akré M. Adja, Franck Remoue, Anne Poinsignon

**Affiliations:** 1 Institut Pierre Richet, Institut National de Santé Publique, Bouaké, Côte d’Ivoire; 2 MIVEGEC, University of Montpellier, CNRS, IRD, Montpellier, France; 3 UFR Sciences de la Nature, Université Nangui Abrogoua, Abidjan, Côte d’Ivoire; 4 UFR Biosciences, Université Félix Houphouët Boigny, Abidjan, Côte d’Ivoire

**Keywords:** *Plasmodium falciparum*, Malaria, Immunity, Salivary proteins, *Anopheles*, Exposure, Immunomodulation

## Abstract

**Background:**

In malaria-endemic areas, human populations are frequently exposed to immunomodulatory salivary components injected during mosquito blood feeding. The consequences on pathogen-specific immune responses are not well known. This study evaluated and compared the humoral responses specific to merozoite stage vaccine candidates of *Plasmodium falciparum*, in children differentially exposed to *Anopheles* bites in a natural setting.

**Methods:**

The cross-sectional study was carried out in Bouaké (Côte d’Ivoire) where entomological data and blood samples from children (0–14 years) were collected in two sites with similar malaria prevalence. Antibody (IgG, IgG1, IgG3) responses to *Pf*AMA1 and *Pf*MSP1 were evaluated by ELISA. Univariate and multivariate analysis were performed to assess the relationship between the immune responses to *P. falciparum* antigens and exposure to *Anopheles* bites in the total cohort and in each site, separately. The individual level of exposure to *Anopheles* bites was evaluated by quantifying specific IgG response to the *Anopheles* gSG6-P1 salivary peptide, which represents a proxy of *Anopheles* exposure.

**Results:**

The anti-*Plasmodium* humoral responses were different according to the level of exposure of children, with those highly exposed to *Anopheles* presenting significantly lower antibody responses to *Pf*MSP1 in total population (IgG and IgG3) and in Petessou village (IgG, IgG1, IgG3). No significant difference was seen for *Pf*AMA1 antigen between children differently exposed to *Anopheles*. In Dar-es-Salam, a neighbourhood where a high *Culex* density was reported, children presented very low antibody levels specific to both antigens, and no difference according to the exposure to *Anopheles* bites was found.

**Conclusion:**

These findings may suggest that immunomodulatory components of *Anopheles* saliva, in addition to other factors, may participate to the modulation of the humoral response specific to *Plasmodium* merozoite stage antigens. This epidemiological observation may form a starting point for additional work to decipher the role of mosquito saliva on the modulation of the anti-*Plasmodium* acquired immunity and clinical protection in combining both field and ex vivo immunological studies.

## Background

Despite the progress achieved in controlling malaria, it remains a major health problem contributing to morbidity and mortality especially in children under 5 years of age in sub-Saharan Africa. Substantial reductions in the global burden of malaria were noted during the past two decades but progress has stagnated. In 2017, the World Health Organization (WHO) estimated that 219 million cases of malaria occurred worldwide, an increase of 2 million from the previous year [1]. Malaria elimination efforts are threatened by the emergence of the resistance of *Plasmodium falciparum* to anti-malarial drugs [2], by increasing and widespread mosquito vector resistance to insecticides [3], and by the lack of an effective vaccine conferring strong protective immunity to infection [4].

In malaria-endemic areas, human populations develop natural immunity against *P. falciparum* that can lead to premunition. This acquired protective immunity takes years to develop after repeated exposure to *Plasmodium* parasite, is relatively short-lived, and is partially effective. It can efficiently control malaria parasite infection leading to a decline in clinical malaria since low parasitaemia mostly persists in the presence of circulating antibodies (Abs). Protective immunity is largely mediated by specific Abs, including immunoglobulin G (IgG) and cytophilic sub-classes (IgG1 and IgG3) [5], that mostly target the *P. falciparum* blood-stage antigens (Ags), such as apical membrane antigen 1 (*Pf*AMA1) [6], merozoite surface protein 1 and 3 (*Pf*MSP1, *Pf*MSP3) [7, 8], and glutamate-rich protein (*Pf*GLURP) [9, 10].

Malaria vaccines currently under development aim to prime such protective responses, particularly in young children and infants. To date, varying formulations of *Pf*AMA1 and *Pf*MSP1 account for the majority of the vaccines that have reached the clinic [11], except the pre-erythrocytic vaccine RTS,S/AS01, based on circumsporozoite protein (CSP) from the sporozoite stage. RTS,S is the most promising malaria vaccine, reaching phase 3 in a clinical trial and approved for use by European regulators in 2015 (Mosquirix™). Modest but significant heterogeneity between individuals regarding the efficacy of infection-blocking vaccine was seen across sites, ranging from 40 to 77% [12].

Immune responses are complex traits and vaccine development requires extensive knowledge of the processes and of the determinants that modulate immune responses in human populations. The effect of age, genetic factors, pathogen co-infection, and nutritional status have been more intensively explored and are recognized to influence anti-*Plasmodium* Ab responses and to have some association with malaria clinical protection [13–15].

Environmental factors as chemical, biological and physical factors may also influence immunity activity [16] and vaccine responses [17]. Environmental exposure can drive epigenetic modification which allows for adaptative immune-T cell activation [18] and memory responses [19] as well as exposure to immuno-toxic or -modulatory activities that can altered immune functions. Interestingly, few studies showed that Ab responses to certain specific vaccines (pneumococcal, rabies, and typhoid vaccines) may be influenced by month of vaccine administration [20] suggesting that seasonally variable environmental Ags may have a co-stimulatory effect on immune responses. Changing temperature, diurnal exposure to sunlight, food availability, and exposure to infectious agents may be part of the different season environmental factors that may act on the immune system. In malaria-endemic areas, human populations are repeatedly exposed to salivary components of blood-feeding mosquitoes that possess a variety of pharmacologically active biomolecules with anti-haemostatic, anti-inflammatory, and immunomodulatory properties to counter the host defense responses activated during a blood meal [21]. There is now evidence that co-injected saliva has immunomodulatory properties, and studies support a role for mosquito saliva in enhancing pathogen transmission via the modulation of Th1/Th2 immune responses [22, 23].

Experimental studies across a wide range of arthropods and their associated pathogens indicated that, generally, insect saliva enhances infection by orientating the immune response of the vertebrate toward a Th2 profile, whereas prior repeated exposure to uninfected bites leads to the development of a Th1 response with a decrease in infection severity [24, 25]. A more recent study in humanized mice suggests that a mixture of Th1 and Th2 responses are upregulated by mosquito saliva and can last for several days in the skin and bone marrow [26]. Most of studies have been performed in murine models and it is obvious that the investigation of this question in human populations from endemic settings is much more complicated. Relatively few studies have investigated the effects of mosquito saliva on human cells ex vivo [27] and on cytokine production from human cells following stimulation with mosquito saliva [28–30]. Altogether, it suggests that human immune response to mosquito saliva is significant and complex: mosquito saliva alters the frequencies of several immune cell populations and cytokine production, in multiple tissues, at several times after blood feeding [26].

The immune microenvironment initiated by arthropod salivary components in the vertebrate host may have consequences for the development of specific immune response against pathogens. So far, few studies have investigated specifically this assumption. Two independent experimental studies suggested that mice exposed either to *Anopheles* or to tick feeding showed a down-regulated Ag-specific immune response compared to naïve mice (model Ag = ovalbumin and BSA, respectively) [31, 32]. Studies extending this approach from murine to natural infection in humans living in malaria-endemic settings are challenging but required. Previous studies showed that acquired anti-*Plasmodium* IgG responses in children differed in two geographic areas where the level of exposure to *Anopheles* vectors was markedly different [33]. Sarr et al. showed a modulation of the balance of cytophilic Ab responses to parasite Ags according to the level of exposure of children to *Anopheles* bites. High exposure to *Anopheles* bites seemed to down-regulate the protective IgG1 Ab responses to whole *Plasmodium* extracts and to CSP Ag, whereas specific IgG3 responses were similar for the two *Plasmodium* Ags in villages exposed to either low or high levels of *Anopheles* bites [34].

The aim of the present study was to evaluate and compare the immunological profiles of IgG, IgG1, and IgG3 responses specific to *Pf*AMA1 and *Pf*MSP1 vaccine candidates, in children differentially exposed to *Anopheles* bites in a natural setting. Interestingly, the intensity of *Anopheles* exposure was assessed at an individual level by evaluating the IgG level specific to the *Anopheles gambiae* gSG6-P1 salivary peptide. For the last decade, new immune-epidemiological tools have been developed that aimed at evaluating the level of exposure to mosquito bites at population and individual level [35]. These innovative tools are based on the measure of human antibody responses to salivary proteins of arthropod vector injected during the bite. As far as the *Anopheles* genus is concerned, the IgG response to the gSG6-P1 peptide (*An. gambiae* Salivary Gland Protein-6 peptide 1) of *An. gambiae* saliva has been identified and validated as a pertinent biomarker of *Anopheles* bites [36–38]. It represents a proxy of human exposure to *Anopheles* bites and is a reliable tool for assessing spatial and temporal heterogeneity of exposure at the individual level [35]. The gSG6-P1 salivary peptide is specific to the *Anopheles* genus, antigenic, easy to synthesize and highly conserved between *Anopheles* mosquitoes.

## Methods

### Ethics statement

The present study followed the ethical principles according to the Helsinki Declaration, and was approved by the Ethics Committee of the Ministry of Health of Côte d’Ivoire (June 2014; No. 41/MSLS/CNER-dkn). Site leaders provided prior permission to survey on each site and informed consent was obtained from all parents or guardians of children.

### Study sites and population

The study was conducted in Bouaké (7° 69 N, 5° 03 W) located in the centre of Côte d’Ivoire. The study area, study design, and local malaria epidemiology have been previously described in detail [39]. Briefly, the climate is tropical humid with two seasons: the dry season runs from November through March, and the rainy season occurs from April to October. The rainy season is marked by two maximum rainfalls, one in June and one in September, with an average annual rainfall of between 1000 and 1600 mm. In this area, malaria transmission is intense with *P*. *falciparum* the major parasite species [40] and *An. gambiaesensu lato* (*s.l.*). the major vector [41].

The initial cohort consisted of 212 children aged from 6 months to 14 years from two sites (a sub-set of a cohort of 508 children from 5 sites [39]) and enrolled in a cross-sectional study which was carried out during the rainy season (August 2014). Households and children were randomly selected and sociological, geographical, entomological, and clinical data were collected. Children’s axillary temperature was measured, and thick films and blood smears were performed for all participants to determine parasite density and *Plasmodium* species. Thick blood smears were fixed and stained with 10% Giemsa and read at double blind by certified microscopists. Asexual parasite densities were counted against 200 microscope fields white blood cells assuming 8000 white blood cells per microlitre. A blood smear was considered negative if no parasites were observed. For quality control, 10% of slides were re-read by blind expert reader.

The present study was carried out on a sub-sample of the initial cohort and consisted of 95 uninfected children aged from 6 months to 14 years from two sites, Dar-es-Salam (a neighbourhood of Bouaké city) and Petessou (a village near Bouaké). Only 76 children were included in the final analysis, after having defined the groups of exposure to *Anopheles* (see below).

For immunological assays, blood samples were collected at the fingertips in microtainer tubes (microvette 500 serum-Gel Starstedt, Marnay, France) and sera were obtained after centrifugation at 3000 rpm for 10 min. Sera were fractionated into aliquots and then frozen at − 20 °C until used.

### Mosquito collection

Adult mosquitoes were collected in June and September 2014, as described [39]. In each of the two sites, six catching points, three indoor and three outdoor were used to collect mosquitoes by landing catches on adult volunteers for two consecutive nights (from 18.00. to 06.00). Adult mosquito catchers gave prior informed consent and received yellow fever vaccination and anti-malarial chemoprophylaxis as recommended by the National Malaria Control Programme. Adult mosquitoes were collected, counted and their species were morphologically classified at the laboratory. The human biting rate (HBR) of each mosquito species was calculated as the average number of mosquitoes collected per person per night.

### Measurement of human IgG antibody level for gSG6-P1 salivary antigen

Human IgG level against the gSG6-P1 salivary antigen of *An. gambiae* was measured by enzyme-linked immunosorbent assay (ELISA). Briefly, 96-well Maxisorp micro-assay plates (Nunc, Roskilde, Denmark) were coated with gSG6-P1 salivary antigen (GPS 1216, Genepep, Saint Jean de Védas, France) at a concentration of 20 μg/mL in phosphate buffered saline (PBS) using 100 µl/well and incubated at 37 °C for 2 h 30. Plates were blocked for 1 h with 200 μL of protein-free blocking buffer, pH7.4 (Thermo scientific, Rockford, USA). The plates were then washed and sera were incubated in duplicate wells at 4 °C overnight at 1/320 dilution in PBS containing 1% of Tween 20 (1%-PBST). Mouse biotinylated Ab to human IgG (BD Pharmingen, San Diego CA, USA) was incubated at a 1/4000 dilution in 1%-PBST (1 h 30 at 37 °C) and Extravidine biotine peroxydase (Amersham, les Ulis, France) was then added (1/20,000; 1 h at 37 °C). Colorimetric development was carried out using ABTS (2.2′-azino-bis (3 ethylbenzthiazoline 6-sulfonic acid) diammonium; Sigma, St Louis, MO, USA) in citrate buffer (Sigma, pH4, containing 0.003% H_2_O_2_) and the optical density (OD) was read 2 h later at 405 nm.

Individual results were expressed as the ΔOD value: ΔOD = ODx-ODn, where ODx represents the mean of individual optical density (OD) value in both wells with gSG6-P1 antigen and ODn the individual OD value for each serum without gSG6-P1 antigen.

Children were separated into 2 groups of exposure according to their IgG level to the gSG6-P1 peptide. The mean value of the total population (ΔOD_gSG6-P1_ mean = 1.25) was determined as the threshold, and individuals (n = 17) presenting ΔOD_gSG6-P1_ mean ± 0.1 (1.15 < OD_gSG6-P1_ < 1.35) have been withdrawn to clearly define 2 groups of individuals differently exposed to *Anopheles*. This results in a ‘low exposure group’ grouping individuals with ΔOD_gSG6-P1_ < 1.15 and a ‘high exposure group’ with individuals with ΔOD_gSG6-P1_ > 1.35, from either Petessou or Dar-es-Salam site.

### Measurement of human IgG, IgG1 and IgG3 antibodies for *Pf*AMA1 and *Pf*MSP1

IgG, IgG1 and IgG3 level to *Pf*AMA1 and *Pf*MSP1 (*Pf*MSP1p_19_) recombinant proteins were measured by indirect ELISA as previously described [9]. Briefly, 96-well Maxisorp micro-assay plates (Nunc, Roskilde, Denmark) were coated with *Pf*AMA1 and *Pf*MSP1 recombinant proteins at a final concentration of 1 µg/ml in coating buffer (PBS with red phenol 0.001%) and incubated at 4 °C overnight. The plates were blocked with skimmed milk buffer (5% milk powder in PBS 0.1% Tween 20 (0.1%-PBST) for 1 h at room temperature. Individual sera were diluted in buffer (1% milk powder in 0.1%-PBST) and added in at a final dilution (1/750 for IgG, 1/200 IgG1 and 1/50 for IgG3) for *Pf*MSP1 and (1/7500 for IgG, 1/3000 IgG1 and 1/100 for IgG3) for *Pf*AMA1. Optimal dilutions were determined after several preliminary experiments. Plates were then incubated for 1 h at room temperature with 100 µl/well of HRP-conjugated goat anti-human IgG (Frederick, USA), IgG1 and IgG3 (Binding Site, Birmingham, UK) diluted respectively for *Pf*MSP1 (1/7500; 1/2000 and 1/1000) and for *Pf*AMA1 (1/5000; 1/2000 and 1/1000) in skimmed milk buffer (1% milk powder in 0.1%-PBST). TMB (Eco Tek, Kuldysen 10, Denmark) was used as a substrate and the reaction was stopped by the addition of 0.2 M H_2_SO_4_ (100 µL/well). The OD was read after 30 min at 450 nm. Individual results (ΔOD) were expressed as above: ΔOD = ODx-ODn, where ODx represents the mean of individual OD value in both wells with *P. falciparum* antigen and ODn the individual OD value for each serum without antigen.

### Data management and statistical analysis

Chi^2^ test was used to compare *Plasmodium* prevalence and HBR between the two studied sites. As antibody levels were not normally distributed, nonparametric tests were used for analyses. A Wilcoxon rank-sum test was used for comparison of Ab levels between two independent groups. Linear regression was performed to compare Ab levels according to age of participants. Generalized linear model (GLM) was used to assess the relationship between the Ab titres specific to *P. falciparum* antigens and covariate factors (age, site and group of exposure). All statistical analysis was done using Prism version 5.0 (Graph Pad Software, San Diego, CA, USA) and R software (Version 3.3.3; R Core Team, Vienna, Austria). All differences were considered as significant at *p* value< 0.05.

## Results

### Study population characteristics, parasitological and entomological data

Population demographic (gender ratio and mean age), parasitological (prevalence and geometric mean of *P. falciparum* density), and entomological data are presented for the initial and the study cohorts, according to the two study sites, in Table 1. In the initial cohort (n = 212), no significant difference was observed in *P. falciparum* prevalence (56.6 and 54%, respectively, χ^2^ = 0.057, df = 1, *p *= 0.811) and in parasite density in infected children (geometric mean (log 10) 3.5 parasites/µl and 4.6 parasites/µl, respectively, *p *= 0.079) between Petessou and Dar-es-Salam sites.

**Table 1:** Characteristics of study population: demographic, parasitological, and entomological data

Site	Dar-es-Salam	Petessou	Total	*p*
Initial cohort				
*n*	99	113	212	–
Thick blood smears				
Negative (%)	43 (43.4)	52 (46.0)	95 (44.8)	0.706
Positive (%)	56 (56.6)	61 (54.0)	117 (55.2)	0.811
Parasite density^a^				
Geometric mean (min–max)	3.55 (2.93–4.29)	4.58 (3.72–5.64)	3.18	0.079
Human biting rate (BHN)				
*An*. g*ambiae*	2	78	–	***
*Culex s.l.*	81	1	–	***
Study cohort^b^				
n	35	41	76	–
Gender ratio (male/female)	0.94 (17/18)	0.95 (20/21)	0.95 (37/39)	0.98
Mean age (years, 95% CI)	6.5 (5.4–7.6)	7.2 (6.3–8.2)	6.9 (5.9–7.9)	0.35
IgG to gSG6-P1^c^				
Low exposure group	0.73 (0.61;0.90)	0.97 (0.74;1.08)	–	0.071
High exposure group	1.84 (1.61;2.11)	1.45 (1.38;1.62)	–	0.001

*n* number of children, *BHN* bites/human/night

^a^Parasite density is expressed as log10 of the number of parasites per µL

^b^Study cohort is composed of uninfected children with ΔOD_gSG6-P1_ < 1.15 or ∆OD_gSG6-P1_ > 1.35

^c^Anti-gSG6-P1 IgG median value (25th; 75th percentile); ***p < 0.001

Table 1 presents the entomological data collected in each study site. The HBR of *An. gambiae* was significantly higher in Petessou than in Dar-es-Salam, with 78 bites per human per night (BHN) and 2 BHN reported, respectively. In Petessou, other *Anopheles* species were captured in much lower proportion: *Anopheles pharoensis* (1.8 BHN), *Anopheles funestus* (0.3 BHN) and *Anopheles welcomei* (0.17 BHN). The HBR of other major nocturnal mosquitoes (*Culex spp.)* was much higher in Dar-es-Salam than in Petessou, with 81 BHN reported in Dar-es-Salam whereas almost no *Culex* was collected in Petessou.

In this study, only the non-infected children residing in the two sites (*n *= 43 from Dar-es-Salam and *n *= 52 from Petessou) were selected. The gender ratio (*p *= 0.98) and mean age (*p *= 0.35) of the sub-set of children were not significantly different between the two study sites (Table 1).

**Fig. 1:**
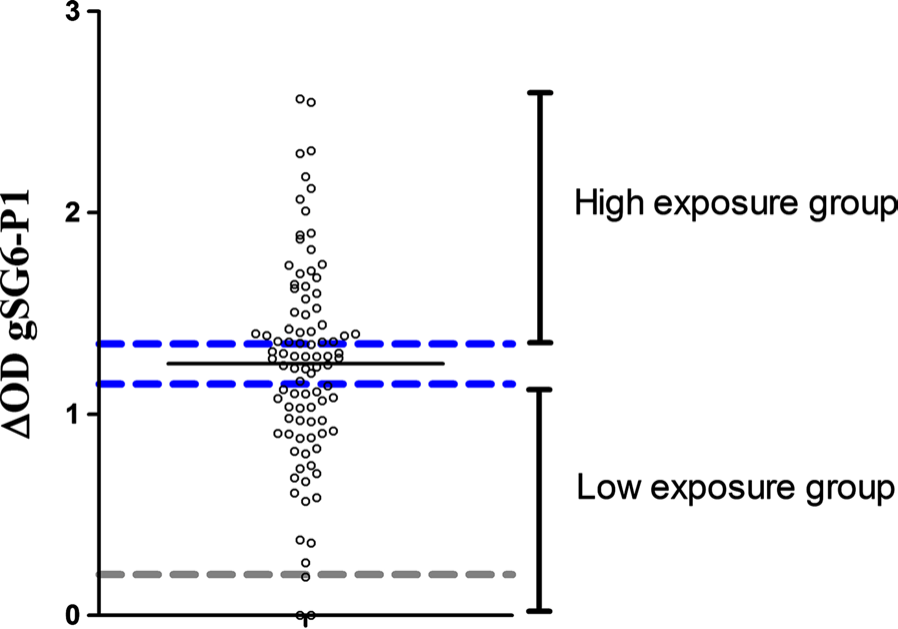
IgG response to *An. gambiae* gSG6-P1 salivary peptide in all uninfected children. Dot plots show the individual specific IgG level to gSG6-P1. Bar indicates the mean value, the grey dot line indicates the cut-off value of seropositivity and the blue dot lines represent the ΔOD_gSG6-P1_ mean ± 0.1 that allow to define the two groups of exposure to *Anopheles* bites. Individuals with ΔOD_gSG6-P1_ < 1.15 were considered as low exposed and individuals with ΔOD_gSG6-P1_ > 1.35 were considered as high exposed to *Anopheles* bites

The specific IgG level to the gSG6-P1 salivary peptide representing a proxy of the intensity of exposure to *Anopheles* bites was also assessed in uninfected children residing in both sites. Children presented a wide range in ∆OD_gSG6-P1_ from 0 to 2.57 (Fig. 1). The median level of IgG response to the gSG6-P1 peptide was similar in children from Dar-es-Salam and Petessou (*p *= 0.129). Children were then separated into low or high exposure group according to their individual ∆OD_gSG6-P1_ value. Children from the low exposure group (∆OD_gSG6-P1_ < 1.15) had a similar IgG median level in Dar-es-Salam and in Petessou sites (*p *= 0.071), which indicated a similar level of exposure to *Anopheles* bites for the children from the low exposure group from each site. On the contrary, children from the high exposure group (∆OD_gSG6-P1_ > 1.35) from Dar-es-Salam had a significantly higher IgG median value than children from Petessou (*p *= 0.001), indicating that children from the high exposure group from Dar-es-Salam were higher exposed to *Anopheles* than did children from Petessou.

### Univariate analysis of specific IgG, IgG1 and IgG3 levels and potential covariate factors

Total IgG, IgG1 and IgG3 responses of the participants to two merozoite (*Pf*AMA1 and *Pf*MSP1) *P. falciparum* stage antigens were evaluated. Univariate analysis was used to investigate the relation between specific anti-*Plasmodium* Ab responses and demographic factors (age, gender) and environmental factors (village and level of exposure to *Anopheles* bites) in the total cohort and in each study site, separately. The results presented in Table 2 showed that the level of the different Ab responses was not significantly (*p *> 0.05) influenced by age or gender.

**Table 2:** Influence of covariate factors on specific IgG, IgG1 and IgG3 antibody levels in children

Covariate factors	Ab response	Total population	*p*	Petessou	*p*	Dar-es-Salam	*p*
Gender (female/male)^a^	IgG anti-*Pf*MSP1	0.16 (0.38;1.12)/0.38 (0.49;1.11)	0.435	0.76 (0.21;2.05)/1.01 (0.19;2.26)	0.835	0.02 (0;0.09)/0.12 (0;0.53)	0.151
	IgG1 anti-*Pf*MSP1	0.14 (0.25;0.92)/0.41 (0.55;1.31)	0.122	0.34 (0.13;1.17)/0.59 (0.30;2.79)	0.07	0.08 (0.01;0.18)/0.13 (0;0.67)	0.58
	IgG3 anti-*Pf*MSP1	0.21 (0.33;0.96)/0.24 (0.40;1.10)	0.322	0.63 (0.24;1.74)/0.65 (0.27;2.70)	0.861	0.00 (0;0.04)/0.03 (0;0.18)	0.065
	IgG anti-*Pf*AMA1	1.02 (0.755;1.563)/0.91 (0.83;1.68)	0.452	1.93 (1.2;2.91)/2.68 (1.28;3.02)	0.506	0.00 (0;0.60)/0.01 (0;0.37)	0.131
	IgG1 anti-*Pf*AMA1	1.19 (1.06;2.15)/0.86 (1.04;2.17)	0.565	3.24 (1.67;3.89)/3.41 (1.86;3.94)	0.758	0.01 (0;0.67)/0.06 (0; 0.58)	0.145
	IgG3 anti-*Pf*AMA1	0.58 (0.55;1.29)/0.37 (0.63;1.49)	0.529	1.15 (0.62;2.34)/1.87 (0.56;3.08)	0.557	0.01 (0; 0.47)/0.034 (0; 0.27)	0.204
Age (years)^b^	IgG anti-*Pf*MSP1	0.002	0.280	− 0.001	0.330	− .031	0.888
	IgG1 anti-*Pf*MSP1	− 0.009	0.551	0.018	0.564	− 0.029	0.788
	IgG3 anti-*Pf*MSP1	0.052	0.287	0.068	0.063	− 0.001	0.335
	IgG anti-*Pf*AMA1	0.001	0.316	− 0.014	0.491	− 0.029	0.825
	IgG1 anti-*Pf*AMA1	− 0.010	0.601	− 0.027	0.878	− 0.029	0.823
	IgG3 anti-*Pf*AMA1	0.029	0.077	0.028	0.150	− 0.018	0.539
Group of exposure *Anopheles* bites (low/high)^a^	IgG anti-*Pf*MSP1	0.53 (0.09;1.97)/0.12 (0.01;0.58)	*0.016*	1.56 (0.76;2.54)/0.27 (0.06;0.67)	*0.001*	0.07 (0;0.16)/0.02 (0;0.55)	0.944
	IgG1 anti-*Pf*MSP1	0.40 (0.10;1.41)/0.16 (0.04;0.50)	*0.034*	1.17 (0.40;2.89)/0.18 (0.13;0.48)	*0.002*	0.10 (0.03;0.27)/0.09 (0;0.87)	1.000
	IgG3 anti-*Pf*MSP1	0.25 (0.05;2.01)/0.11 (0.00;0.48)	*0.040*	1.29 (0.41;2.72)/0.45 (0.23;1.14)	*0.030*	0.02 (0; 0.12)/0.01 (0; 0.10)	0.505
	IgG anti-*Pf*AMA1	1.00 (0.04;2.74)/0.80 (0;2.57)	0.345	1.58 (1.17;3.01)/2.50 (1.37;2.88)	0.864	0.03 (0.02;0.14)/0 (0; 0.65)	0.285
	IgG1 anti-*Pf*AMA1	0.78 (0.07;3.26)/0.98 (0.01;3.43)	0.456	2.70 (1.56;3.95)/3.51 (2.54;3.89)	0.368	0.06 (0;04; 0.283)/0.01 (0;0.79)	0.216
	IgG3 anti-*Pf*AMA1	0.79 (0.04;2.62)/0.17 (0.0;1.16)	0.076	2.01 (0.78;3.03)/0.93 (0.50;2.07)	0.124	0.02 (0.0; 0.29)/0.01 (0;0.43)	0.533
Site (Petessou/Dar-es-Salam)^a^	IgG anti-*Pf*MSP1	0.80 (0.21;2.20)/0.05 (0;0.22)	<* 0.0001*				
	IgG1 anti-*Pf*MSP1	0.45 (0.18;2.12)/0.09 (0;0.27)	< *0.0001*				
	IgG3 anti-*Pf*MSP1	0.64 (0.26;1.83)/0.01 (0;0.12)	<* 0.0001*				
	IgG anti-*Pf*AMA1	2.47 (1.25;2.97)/0.01 (0;0.18)	<* 0.0001*				
	IgG1 anti-*Pf*AMA1	3.33 (1.71;3.91)/0.02 (0.01;0.21)	<* 0.0001*				
	IgG3 anti-*Pf*AMA1	1.52 (0.61;2.8)/0.02 (0;0.05)	<* 0.0001*				

^a^Median (25th; 75th percentile)

^b^Adjusted R-squared

The comparison of the Ab responses between groups of exposure indicated that a higher exposure to *Anopheles* mosquitoes was associated with a lower Ab levels to *Pf*MSP1 antigen in the total population and in Petessou, but not in Dar-es-Salam. Indeed, children from the high exposure group had significantly lower IgG, IgG1 and IgG3 responses specific to *Pf*MSP1 compared to children from the low exposure group in total population (*p *= 0.016, *p *= 0.034 and *p *= 0.04, respectively) and in Petessou (*p *= 0.001, *p *= 0.002 and *p *= 0.03, respectively). In contrast, difference in *Anopheles* exposure was not associated with a statistically significant effect on the levels of Ab responses to *Pf*AMA1 antigen in the total population as well as in each site.

The comparison of Ab responses between site showed children from Dar-es-Salam presented significant lower Ab levels to *Pf*AMA1 and *Pf*MSP1 *P. falciparum* Ags compared to children from Petessou (all *p *< 0.0001). In Dar-es-Salam, the median values of Ab responses specific to both Ags were very low.

### Multivariate analysis of specific IgG, IgG1 and IgG3 responses in total population and in each study site

A multivariate analysis was used to further assess the relationship between the immune responses to *P. falciparum* merozoite stage antigens and exposure to mosquito bites. Age, level of exposure (group of exposure) and site were used as predictor variables (Table 3). In a general pattern, children from Dar-es-Salam presented significantly lower Ab responses to *Pf*AMA1 and *Pf*MSP1 than children from Petessou (all *p *< 0.01) and no association was found between Ab responses to *P. falciparum* antigens and age. In the total population, a negative association was observed between the level of exposure and the anti-*Pf*MSP1 Ab titres. Indeed children from the high exposure group presented a significant lower anti-*Pf*MSP1 IgG (− 0.443, *p *= 0.043) and IgG3 (− 0.422, *p *= 0.033) titres whereas no association was found between Ab responses to *Pf*AMA1 and groups of exposure. The same trend was observed only for children from Petessou, when the analysis was restricted to site. A higher exposure to *Anopheles* mosquitoes was associated with a trend toward decreased of the IgG (− 1.02, *p *= 0.003), IgG1 (− 1.09, *p *= 0.006) and IgG3 (− 0.653, *p *= 0.047) responses to *Pf*MSP1 antigen only.

**Table 3:** Multivariate analysis of specific IgG, IgG1 and IgG3 antibody response levels in children

Ab response	Covariate factors	Total population		Petessou		Dar-es-Salam	
		Estimate	*p*	Estimate	*p*	Estimate	*p*
IgG anti-*Pf*MSP1	Constant	1.240		1.488		0.172	
	Age	0.018	0.58	0.069	0.715	− 0.005	0.871
	Group of exposure (ref = low exposure)	− 0.443	*0.043*	− 1.02	*0.003*	0.268	0.232
	Site (ref = Petessou)	− 0.823	< *0.001*				
IgG1 anti-*Pf*MSP1	Constant	1.230		1.623		0.245	
	Age	0.006	0.883	− 0.004	0.943	− 0.012	0.753
	Group of exposure (ref = low exposure)	− 0.397	0.107	− 1.09	*0.006*	0.386	0.148
	Site (ref = Petessou)	− 0.66	*0.008*	–	–	–	–
IgG3 anti-*Pf*MSP1	Constant	0.951		0.881		0.081	
	Age	0.057	0.066	0.081	0.154	0.027	0.363
	Group of exposure (ref = low exposure)	− 0.422	*0.033*	− 0.653	*0.047*	− 0.126	0.496
	Site (ref = Petessou)	− 0.895	< *0.0001*	–	–	–	–

Ab response	Covariate factors	Total population		Petessou		Dar-es-Salam	
		Estimate	*p*	Estimate	*p*	Estimate	*p*
IgG anti-*Pf*AMA1	Constant	− 0.025		1.64		0.129	
	Age	0.018	0.562	0.044	0.416	− 0.007	0.813
	Group of exposure (ref = low exposure)	0.196	0.324	0.222	0.506	0.211	0.311
	Site (ref = Petessou)	− 1.871	< *0.0001*	–	–	–	–
IgG1 anti-*Pf*AMA1	Constant	0.028		2.37		0.216	
	Age	0.006	0.87	0.029	0.683	− 0.008	0.813
	Group of exposure (ref = low exposure)	0.379	0.125	0.557	0.201	0.216	0.366
	Site (ref = Petessou)	− 2.576	< *0.0001*	–	–	–	–
IgG3 anti-*Pf*AMA1	Constant	0.021		1.42		0.045	
	Age	0.046	0.167	0.069	0.262	0.014	0.548
	Group of exposure (ref = low exposure)	− 0.279	0.198	− 0.525	0.174	0.053	0.724
	Site (ref = Petessou)	− 1.462	< *0.0001*	–	–	–	–

## Discussion

Evidence now suggested that arthropod vectors on top of transmitting pathogens may also have roles in facilitating transmission and influencing disease evolution [25]; for instance the immuno modulatory properties of the co-injected saliva acts both on innate and adaptative immune responses of the vertebrate host [42]. Host immune responses to arthropod saliva are varied and complex, and depend both on the host and vector species [43]. The present study aimed to investigate the relationships between specific Ab responses to merozoite stage antigens (*Pf*MSP1 and *Pf*AMA1) in children differently exposed to *Anopheles* bites in two study sites in Côte d’Ivoire. Results showed that children higher exposed to *Anopheles* bites presented lower IgG, IgG1 and IgG3 responses to *Pf*MSP1 in Petessou. No association between the level of exposure and Ab responses to *Pf*AMA1 antigen was observed.

Exposure to *Anopheles* bites was investigated via two complementary methods: at the site scale with entomological indicators (human landing catches (HLC)) and at the individual level by using a serological biomarker of exposure based on the quantification of the IgG response specific to the *An. gambiae* gSG6-P1 salivary peptide. The two approaches did not give the same level of information about the exposure to *Anopheles* bites. HLC method indicates the mean number of bites that an individual may receive per night. Thus, it appreciates as an approximate proxy the level of exposure for each *Culicidae* species at site scale but does not take into account the inter-individual heterogeneity of exposure in natural setting. Indeed, environmental factors generating hot-spots of exposure (proximity to breeding sites for example), attraction an individual exerts on mosquitoes and the use of personal protection against mosquito bites (such as nets and coils) suggest that exposure to *Anopheles* bites can be highly variable from house to house and also between people living in the same house. Only the serological approach reflects the human–*Anopheles* contact and integrates the individual risk factors of being bitten. It provides for each participant a proxy of the individual level of exposure to *Anopheles* bites and thus, is more appropriate for reflecting the inter-individual heterogeneity of exposure in natural setting. Numerous studies have evidenced the *Anopheles* gSG6-P1 salivary peptide represents a reliable and complementary tool to entomological methods for assessing spatial and temporal heterogeneity of exposure to *Anopheles* bites at the individual level [35].

On the basis of comparison of HBR, children from Petessou had significantly higher exposure to *Anopheles* bites than did children from Dar-es-Salam, whereas the comparison of anti-gSG6-P1 IgG level (ΔOD_gSG6-P1_ median and range) suggested that exposure to *Anopheles* bites was similar between the two sites. In Dar-es-Salam, the low number of *Anopheles* female caught during the entomological surveys may be explained by a low availability of breeding sites for *Anopheles* in urban setting. *Anopheles* classically like small, open, sunlit, fresh stagnant water suggesting a mosaic of isolated pockets of *Anopheles* breeding sites in the urban context that may result of local hot spots of *Anopheles* exposure that could not have been identified. As mentioned above, the use of vector control strategies and/or sociological factors specific to the urban context may also explain the discrepancies observed between the two approaches [39]. Parasitological data indicated that *Plasmodium* prevalence and density (the gold standard to measure the transmission of malaria) were similar between the two sites, thereby suggesting that children were exposed similarly to malaria transmission.

According to their individual anti-gSG6-P1 IgG level, children were separated into two exposure groups: low and high group of exposure to *Anopheles* bites. In the present study, when applying the cut-off of positivity (ΔOD_gSG6-P1_ = 0.204) [44, 45], one individual from Petessou and two from Dar-es-Salam were seronegative indicating that all but three individuals can be considered exposed to *Anopheles*. In addition, the wide range of ΔOD_gSG6-P1_ values in either Petessou and Dar-es-Salam, suggested that participants with higher ΔOD_gSG6-P1_ values were bitten more in comparison to those with lower ΔOD_gSG6-P1_ values and thereby applying a threshold (mean ΔOD_gSG6-P1_ ± 0.1) would stratified the population in two groups with different level of exposure to *Anopheles* bites.

IgG, IgG1 and IgG3 levels to *Pf*AMA1 and *Pf*MSP1 merozoite stage antigens were compared according to demographic factors and between groups of exposure. No statistically significant effect of age (univariate and multivariate analysis) or gender (univariate analysis) on the level of Abs specific to *Pf*AMA1 and *Pf*MSP1 was noted. According to univariate and multivariate analysis, specific Ab titres to *Pf*MSP1 differed significantly between groups of exposure in total population (IgG and IgG3) and in Petessou village (IgG, IgG1 and IgG3). A higher exposure to *Anopheles* bites was associated with a significant trend toward lower IgG, IgG1 and IgG3 responses specific to *Pf*MSP1. A higher Ab titres could be expected in children higher exposed to *Anopheles* bites and, therefore, to malaria transmission. Nevertheless, the epidemiological observation in the present study is consistent with studies that reported a down-regulated immune response to specific Ag in mice exposed to arthropod saliva compared to naïve mice [31, 32]. Exposure to biting mosquitoes may lead to a modification of the host’s immune cells and of the balance between Th1 and Th2 cytokine production [26]. Several experimental murine studies showed that Th2 cytokines as IL-4, the inhibitory cytokine IL-10 and the pro-inflammatory cytokine TNF, were up-regulated after uninfected *Aedes, Culex* or *Anopheles* mosquito bites [31, 46, 47]. The secretion of the immunosuppressive IL-10 cytokine could increase the proliferation of T regulatory cells and down-regulate specific Ab immune response because it inhibits Ag presentation, IFN-γ expression, and macrophage activation. IL-10 has also been involved in the balance of cytophilic Ab responses [48]. Thus, it is conceivable that the potential decrease of Ab levels in children higher exposed may be associated with a lower IL-10 production. This hypothesis could be assessed by analysing the ex vivo cytokine production of immune cells from individuals differently exposed to *Anopheles* bites.

Only a few epidemiological studies have previously reported an association between exposure to *Anopheles* immunomodulatory saliva and acquisition of natural immunity to *Plasmodium* in natural settings. Sarr et al. reported that children with higher exposure to *Anopheles* bites presented a down-regulated IgG1 response to whole *Plasmodium* extract and to CSP Ag compared to children lower exposed, whereas no effect was observed for the IgG3 isotype response [34]. Dechavanne et al. reported that an environmental variable (quantitative index related to the spatiotemporal risk of exposure to *Anopheles* mosquitoes) was significantly associated with high anti-*Plasmodium* Ab levels in infants (6–18 month old infants) [13]. A recent study in malaria elimination context also showed a positive association between *Anopheles* exposure and IgG responses to *Pf*CSP and *Pf*MSP1 Ags [49]. Differences observed between studies might be attributed to the different context of exposure to *Anopheles* bites. Indeed, the history and intensity of exposure to mosquito bites may have different effect on immune system, as mentioned in experimental studies that reported a immunostimulatory effect with low concentrations of saliva whereas high concentrations were immunosuppressive [50]. Human studies in different malaria context are therefore needed even if challenging, due to numerous co-factors from the parasite, the human-host or environment that may modulate human immune system. The present study was carried out in uninfected children in order to minimize the antigenic boost of recent infection, thus the time since previous *P. falciparum* infection and rates of antibody decay may also participate in the differences observed between individuals from different groups of exposure. This represents a limit to the present study in addition to the small sample size. Malaria prevalence was near 50% in Petessou, thus it could be expected that individuals were regularly infected by *Plasmodium* parasites and that their last infection, symptomatic or asymptomatic, was recent. Other factors such as genetic background, co-infections, or nutritional status may also explain some of the differences in the immunological profiles observed between the two exposure groups.

No association between groups of exposure to *Anopheles* bites and humoral responses (IgG, IgG1 and IgG3) to *Pf*AMA1 merozoite stage Ag was showed. This difference of effect according to *Plasmodium* Ags may be due to different intrinsic characteristics to merozoite Ags.

Children from Dar-es-Salam presented low level of anti-*Plasmodium* Abs. Parasitological data indicated that both sites had a similar intensity of malaria transmission with around 50% of malaria prevalence in the initial population. This suggests that factors other than parasite exposure alone may modulate anti-*Plasmodium* humoral responses. For example, the effect of human genetic factors cannot be excluded since the study children did not belong to the same ethnic group [51, 52]. The population from Dar-es-Salam is mostly composed of Dioula and Manding, whereas autochthonous Baoulé live in the rural village of Petessou. Mosquito saliva is known to contain close to 100 secretory proteins, and comparative analyses indicated that *Culex* and *Aedes* saliva have specific salivary proteins not found in *Anopheles* saliva [53, 54]. The exposure to other mosquito salivary components with immunomodulatory properties may also participate in the modulation of the anti-*Plasmodium* immune responses. The presence of anthropophilic *Culex* species was reported only in Dar-es-Salam site by entomological data. Exposure to immunomodulatory components of *Culex* saliva may also contribute, in addition to other factors, to the down-regulation of the immune responses observed in children from Dar-es-Salam. The availability of a serological biomarker of exposure assessing specifically the exposure to *Culex spp.* bites at the individual level would be valuable in multivariate analysis to strengthen the hypothesis.

Further studies are needed in order to better characterize the effects of mosquito saliva on human immune system, for example by analysing ex vivo cytokine production after stimulation of peripheral blood of mononuclear cells from individuals differentially exposed to mosquito bites. More evidence on its immunomodulatory effects on naturally acquired immunity to *Plasmodium* has to be provided with field studies in different epidemiological context.

Immune responses are complex traits and little is known about the environmental factors modulating acquired immunity to *Plasmodium* and premunition despite its essential role in the clinical outcome of malaria infections and in the development of vaccine immunity. Salivary modulators of the immune system could be prime targets for the development of transmission-blocking vaccines. Indeed, taking advantage of the modulation induced by saliva (e.g., neutralization of the immune suppression) would help the host’s immune system to respond to pathogens. Interestingly, over the past few years, combining pathogen and salivary Ags in a single vaccine is seen as a valuable option [55]. Elucidating the mechanism may also lead to the discovery of new immunosuppressive molecules of therapeutic interest. These findings may also have an important impact on the evaluation of vaccine efficacy. Inter-individual variability in humoral immune responses to a specific *P. falciparum* antigen has been reported in different studies evaluating the immunogenicity of vaccine candidates [56, 57]. The exposure to immunomodulatory insect salivary proteins during or after the immunization period could modulate the acquisition as well the durability of Ab responses to vaccine Ag, as noted for rabies or typhoid vaccines [20]. It could also have consequences on the immunological profiles induced in terms of intensity and/or isotype distribution, and thus on the efficacy of the protective immunity. In this framework, it is clear that a better understanding of the modulation of protective and anti-vaccine immune responses by epidemiological and environmental factors is of public health relevance and would be valuable for malaria vaccine development.

## Conclusion

The main results of the present study show children differently exposed to *Anopheles* bites presented different levels of Ab responses to *Pf*MSP1 antigen. These findings may suggest that immunomodulatory components of *Anopheles* saliva, in addition to other factors, may participate to the modulation of the humoral response specific to *Plasmodium* merozoite stage antigens. This epidemiological observation may form a starting point for additional works to better evidence and characterize the effects of mosquito saliva on the human immune system and on the anti-*Plasmodium* acquired immunity in combining both field and ex vivo immunological studies.

## Data Availability

The dataset analysed during the current study is available from the last author on reasonable request.

## Abbreviations

gSG6-P1: *An. gambiae* Salivary Gland Protein-6 peptide 1
IgG: Immunoglobulin G
ELISA: Enzyme-linked immunosorbent assay
ΔOD: Delta optical density
*Pf*AMA1: *Plasmodium falciparum* apical membrane antigen 1
*Pf*MSP1: *Plasmodium falciparum* merozoite surface protein 1
HBR: Human biting rate
BHN: Bites per human per night
